# Enhancing systemic resistance in faba bean (*Vicia faba* L.) to *Bean yellow mosaic virus via* soil application and foliar spray of nitrogen-fixing *Rhizobium leguminosarum* bv. *viciae* strain 33504-Alex1

**DOI:** 10.3389/fpls.2022.933498

**Published:** 2022-08-02

**Authors:** Ahmed Abdelkhalek, Hamada El-Gendi, Abdulaziz A. Al-Askar, Viviana Maresca, Hassan Moawad, Mohsen M. Elsharkawy, Hosny A. Younes, Said I. Behiry

**Affiliations:** ^1^Department of Plant Protection and Biomolecular Diagnosis, Arid Lands Cultivation Research Institute (ALCRI), City of Scientific Research and Technological Applications, Alexandria, Egypt; ^2^Department of Bioprocess Development, Genetic Engineering and Biotechnology Research Institute, City of Scientific Research and Technological Applications, Alexandria, Egypt; ^3^Department of Botany and Microbiology, College of Science, King Saud University, Riyadh, Saudi Arabia; ^4^Department of Biology, University of Naples “Federico II”, Naples, Italy; ^5^Department of Agricultural Microbiology, National Research Centre, Cairo, Egypt; ^6^Department of Agricultural Botany, Faculty of Agriculture, Kafrelsheikh University, Kafr El-Sheikh, Egypt; ^7^Department of Agricultural Botany, Faculty of Agriculture (Saba Basha), Alexandria University, Alexandria, Egypt

**Keywords:** *Rhizobium leguminosarum* bv. *viciae*, *Bean yellow mosaic virus*, faba bean, induced systemic resistance, oxidative stress, pathogenesis-related proteins, HPLC, GC-MS

## Abstract

*Rhizobium* spp. manifests strong nitrogen fixation ability in legumes. However, their significance as biocontrol agents and antivirals has rarely been investigated. Under greenhouse conditions, the molecularly identified nitrogen-fixing plant growth-promoting rhizobacteria (PGPR), *Rhizobium leguminosarum* bv. *viciae* strain 33504-Alex1, isolated from the root nodules of faba bean plants, was tested as a soil inoculum or a foliar application to trigger faba bean plants’ resistance against *Bean yellow mosaic virus* (BYMV) infection. Compared to the non-treated faba bean plants, the applications of 33504-Alex1 in either soil or foliar application significantly promoted growth and improved total chlorophyll content, resulting in a considerable reduction in disease incidence and severity and the inhibition index of BYMV in the treated faba bean plants. Furthermore, the protective activities of 33504-Alex1 were associated with significant reductions in non-enzymatic oxidative stress markers [hydrogen peroxide (H_2_O_2_) and malondialdehyde (MDA)] and remarkably increased DPPH free radical scavenging activity and total phenolic content compared to the BYMV treatment at 20 days post-inoculation. Additionally, an increase in reactive oxygen species scavenging enzymes [superoxide dismutase (SOD) and polyphenol oxidase (PPO)] and induced transcriptional levels of pathogenesis-related (PR) proteins (*PR-1*, *PR-3*, and *PR-5*) were observed. Of the 19 polyphenolic compounds detected in faba bean leaves by high-performance liquid chromatography (HPLC) analysis, gallic and vanillic acids were completely shut down in BYMV treatment. Interestingly, the 33504-Alex1 treatments were associated with the induction and accumulation of the most detected polyphenolic compounds. Gas chromatography-mass spectrometry (GC-MS) analysis showed hexadecanoic acid 2,3-dihydroxypropyl ester, tetraneurin-A-Diol, oleic acid, and isochiapin B are the major compounds in the ethyl acetate extract of 33504-Alex1 culture filtrate (CF), suggesting it acts as an elicitor for the induction of systemic acquired resistance (SAR) in faba bean plants. Consequently, the capacity of *R. leguminosarum* bv. *viciae* strain 33504-Alex1 to enhance plant growth and induce systemic resistance to BYMV infection will support the incorporation of 33504-Alex1 as a fertilizer and biocontrol agent and offer a new strategy for crop protection, sustainability, and environmental safety in agriculture production.

## Introduction

A sustainable increase in the human population and the global reduction in the agricultural area force the requirement for higher plant yields (per unit area) with enhanced quality (Shah et al., 2021). However, extensive agriculture for food supplementation and the toxic negative effect of industrial discharge are challenged by numerous milestones that restrict the continuous food supply and result in significant financial losses (Abd El-Rahim et al., 2016; Saeed et al., 2021). Plant viral infection is a dominant agriculture challenge that adversely affects yield and quality (Abdelkhalek and Hafez, 2020). Some plant viruses have a broad host range and multiple transmission mechanisms that complicate the control process and account for significant losses in several crops (Scholthof et al., 2011; Adhikari et al., 2021). *Bean yellow mosaic virus* (BYMV) is one of the most noteworthy plant viruses that affect more than 60 plant species in legumes and non-legumes (Chatzivassiliou, 2021). In addition, the various modes of viral transmission make BYMV a major economic threat to crop production. BYMV-infected beans are associated with mosaic, necrosis, and mottle symptoms. It can be spread by aphids, seeds, and mechanical inoculation (Ismail and Atef, 1998; El-Helaly et al., 2016).

Among the other Fabaceae members, the faba bean (*Vicia faba* L.) is an essential and economically important plant, severely affected by BYMV infection that directly affects the crop yield (Chatzivassiliou, 2021). Due to the highly beneficial and nutritional values of faba bean grains, they are directly utilized in the human diet in the Middle East and East Asia countries and are applied in animal feeding recipes worldwide (Duc et al., 2010; Adhikari et al., 2021). The protein content of faba bean grains ranged from 26 to 41%, representing an ideal protein source for plant-based diets (Fernández et al., 1996; Warsame et al., 2020). Despite the crop’s economic importance, its cultivation area was globally reduced to 2.6 million hectares in 2019, compared to 5.6 million hectares in 1961 (Adhikari et al., 2021). Crop losses due to faba bean sensitivity to biotic and abiotic stresses could partially explain this decline. It was found that BYMV infection caused a decrease in leaf area and flower production, as well as yellowing, mosaicing, mottling, and bending of plants, which could cause crop losses of up to 30% (Moury and Desbiez, 2020).

Developing new resistant plants and/or controlling the viral transmission vector as best as possible are more applicable approaches (Elbadry et al., 2006). Though developing new resistance cultivars through genetic engineering is an important step toward overcoming plant infection in the field, the infectious agent may manage to overcome this resistance and complicate the problem through resistance spreading (Melero-Vara et al., 2000). On the other hand, the widespread use of chemical pesticides exacerbates environmental issues by accumulating hazardous waste and negatively impacting crop quality and human health (Vejan et al., 2016). Increasing plant systemic immunity is an eco-friendly and more effective approach for plant viral control. Plant growth-promoting rhizobacteria (PGPR) have improved plant growth under various biotic and abiotic stresses (Al-Ani and Adhab, 2013; Karavidas et al., 2022; Rizvi et al., 2022). The PGPRs produce a wide range of bioactive chemicals that could induce the plant systemic resistance by activating many plant protection genes against viruses and/or enhancing the plant growth for better nutrient and water acquisition (Vejan et al., 2016; Abdelkhalek et al., 2020a,b).

Biological nitrogen fixation is a vital process in agriculture since it allows for nitrogen production through legume-rhizobia symbiosis, which contributes to rising nitrogen levels in the soil, resulting in enhanced plant growth (Moawad et al., 2005). The direct and indirect augmentation of plant growth and immunity by PGPR renders plants more resistant to surrounding stresses and infections (Saeed et al., 2021). Rhizobia, one of the PGPRs, has demonstrated a high level of nitrogen fixation in legumes and stimulating plant defense mechanisms. Al-Ani and Adhab (2013) and Elhelaly (2022) found that BYMV infection decreased root and shoot dry weights, while rhizobia inoculation increased these parameters significantly. Nonetheless, their potential as biocontrol agents, particularly antiviral agents, has received little attention. Therefore, this study aimed to evaluate the ability of *Rhizobium leguminosarum* bv. *viciae* strain 33504-Alex1 to promote faba bean growth and boost its resistance to BYMV infection either through soil inoculation or foliar application of culture filtrate (CF) to the plant leaves. Moreover, non-enzymatic oxidative stress markers [hydrogen peroxide (H_2_O_2_) and malondialdehyde (MDA)], DPPH free radical scavenging activity, total phenolic content, and reactive oxygen species scavenging enzymes [superoxide dismutase (SOD), catalase (CAT), ascorbate peroxidase (APX), and polyphenol oxidase (PPO)] were assessed. In addition, the expression of four faba bean pathogenesis-related (PR) proteins (*PR-1*, *PR-2*, *PR-3*, and *PR-5*) was also evaluated. Additionally, high-performance liquid chromatography (HPLC) and gas chromatography-mass spectrometry (GC-MS) analysis were used to figure out the metabolites in faba bean plant extract and the bioactive constituents of 33504-Alex1-CF.

## Materials and methods

### Viral isolation, identification, and molecular characterization

Faba bean (*Vicia faba* L.) leaves showing BYMV-like symptoms were collected from an open field in the Alexandria governorate in 2021. The samples were checked for virus presence using a complete double antibody-sandwich enzyme-linked immunosorbent assay (DAS-ELISA) kit (DSMZ, RT-0717) as described previously (Clark and Adams, 1977). Infected faba bean leaves that reacted positively to DAS-ELISA were mechanically inoculated on *Chenopodium amaranticolor* leaves to induce chlorotic local lesion signs (Younes et al., 2021), which were later used as a source for BYMV. The Plant Virus RNA Kit PVR050 (Geneaid Biotech Ltd., New Taipei City, Taiwan) was used to extract viral RNA from faba bean plants. A 2 g of DNase I-treated RNA was reverse-transcribed into cDNA using Maxima Reverse Transcriptase (Thermo Fisher Scientific, Waltham, MA, United States). As previously described, a PCR reaction was carried out with 2 μl of generated cDNA and BYMV-CP gene-specific primers (Table 1; Heflish et al., 2021). The PCR product was examined for specificity on a 1.5% agarose gel electrophoresis, purified with a PCR clean-up column kit, and sent for sequencing. NCBI-BLAST^1^ compared the annotated nucleotide sequences to sequences of previously reported BYMV isolates. The annotated sequences were then deposited in GenBank and given an accession number.

**TABLE 1:** Nucleotide sequences of primers used in this study.

Primer name	Abbreviation	Direction	Nucleotide sequence
*Bean yellow mosaic virus*-coat protein	BYMV-CP	Forward	GGTTTGGCYAGRTATGCTTTTG
		Reverse	GAGAATTTAAAGACGGATA
16S ribosomal RNA	16S rRNA	Forward	AGAGTTTGATCCTGGCTCAG
		Reverse	GGTTACCTTGTTACGACTT
Pathogenesis related protein-1	*PR-1*	Forward	GTTCCTCCTTGCCACCTTC
		Reverse	TATGCACCCCCAGCATAGTT
Endoglucanase	*PR-2*	Forward	TATAGCCGTTGGAAACGAAG
		Reverse	CAACTTGCCATCACATTCTG
Chitinase	*PR-2*	Forward	ATGGAGCATTGTGCCCTAAC
		Reverse	TCCTACCAACATCACCACCA
Thaumatin-like protein	*PR-5*	Forward	AATTGCAATTTTAATGGTGC
		Reverse	TAGCAGACCGTTTAAGATGC
β-actin	β-actin	Forward	TGGCATACAAAGACAGGACAGCCT
		Reverse	ACTCAATCCCAAGGCCAACAGAGA

### Bacterial isolation, characterization, and molecular identification

The *Rhizobium* isolates applied in the current work were locally isolated from faba bean root nodules collected from an open field in Alexandria governorate (Vincent, 1970). The isolates were tested for symbiotic effectiveness with faba bean plants cv. Giza 716 as described by Somasegaran and Hoben (2012). The best bacterial isolate was characterized and molecularly identified using the 16S rRNA sequencing methodology. The bacterial genomic DNA was extracted according to the manufacturer’s instructions of the Wizard Genomic DNA Purification Kit (Promega Corporation, WI, United States). The full-length 16S rRNA gene was subjected to PCR amplification using the universal 16S rRNA primers (Table 1). The purified 16S rRNA gene was subjected to identification using an automated DNA sequencer (ABI PRISM model 310, Sigma Scientific Services Company, United States). The resulting sequence was aligned through CLUSTALW (1.82) and compared with sequences in the GenBank database through BLASTN search tools at the National Center for Biotechnology Information site.^2^ The MEGA program generated the phylogenetic relationship (ver. 11) based upon the bootstrap neighbor-joining tree from the CLUSTALW alignment.

### Inoculum preparation and experimental greenhouse design

A pure colony of the *Rhizobium* isolate was inoculated in 100 ml of Yeast Extract Mannitol (YEM) broth media. The culture was grown at 28°C and shaken at 150 rpm for 48 h until it achieved 10^9^ CFU/ml. On the other hand, the CF was obtained after spinning the bacterial culture at 10,000 rpm for 10 min. A pot experiment was conducted to evaluate the efficiency of the isolated *Rhizobium* isolate and its secondary metabolites for inducing systemic resistance of faba bean plants against BYMV. A virus-free seed of faba bean cultivar Giza 716 was obtained from the Agriculture Research Center, Egypt. After being surface-sterilized, the uniform seeds were grown in 40 cm plastic pots containing a sterilized equal mixture of clay, sand, and peat moss. Plants of similar sizes were chosen after 17 days of growth and distributed into four treatment groups. The first treatment (control group, G1) was faba bean plants inoculated with viral inoculation buffer and foliar sprayed with sterile non-inoculated broth medium. The second treatment (virus group, G2) was a group of plants mechanically inoculated with BYMV and foliar sprayed with a sterile non-inoculated broth medium. The third treatment (soil group, G3) includes soil inoculated plants 4 days before BYMV inoculation with the *Rhizobium* isolate. Each pot was inoculated with 2 ml of the culture having 10^9^ CFU/ml. The fourth treatment (foliar group, G4) was plants treated by foliar spraying CF 24 h before inoculation with BYMV. The CF was sprayed on the whole plant shoots until the leaves looked like they were covered with it. Each treatment contained five biological replicates. Each of which consisted of a pool of 9 faba bean leaves collected from the three plants in each pot (3 leaves/plant). Each biological replicate was run in three technical replicates. The BYMV inoculation was done on the 21st day of the growth. Briefly, each plant’s two true upper leaves were carborundum-dusted (600 mesh) and mechanically inoculated with BYMV as described previously (Abdelkhalek et al., 2022). All plants were kept under insect-proof greenhouse conditions of 28/16°C (day/night) and 70% relative humidity and were observed daily for symptom development.

### Disease assessment and detection of *Bean yellow mosaic virus* by enzyme-linked immunosorbent assay

At 20 days post-viral inoculation (dpi), faba bean plants were harvested and evaluated in terms of plant shoot and root growth parameters and total chlorophyll content directly measured in the plant leaves through the Chlorophyll Meter SPAD-502Plus (Konica Minolta, Inc., Tokyo, Japan). Moreover, enzyme activity estimation and HPLC analysis were also estimated simultaneously. The DAS-ELISA complete kit (DSMZ, RT-0717) was used to detect BYMV and measure its concentrations within the inoculated tissues. ELISA was performed on both BYMV-challenged inoculated and non-inoculated leaves (to check systemic virus movement) of faba bean plants. The absorbance value of A405 of the sample was reported to be positive for the presence of viruses if it was double the threshold value of the healthy control samples. The viral inhibition index was measured according to El Gamal et al. (2022) as follows:


(1) $$\mathrm{Viral}\mathrm{inhibition}\mathrm{index}\left(\%\right)=\frac{\text{C}-\text{T}}{\text{C}}\times 100$$

where “C” represents the mean average A_405_ value of untreated positive control plants (virus group, G2) and “T” represents the average A_405_ value of each treatment group (G3 and G4).

Disease incidence was calculated using the formula: Disease incidence (%) = Total number of infected plants/total number of plants × 100 (El Gamal et al., 2022). The disease severity (DS) was recorded using the rating scale: 0 indicates no symptoms, 1 indicates chlorotic local lesions and mild mosaic, 2 indicates severe mosaic, and 3 indicates malformation. Disease severity values were calculated using the following formula (Radwan et al., 2008).


(2) $$\text{DS}\left(\%\right)=\frac{\begin{array}{c} \text{Σ}(\mathrm{disease}\mathrm{scale}\times \mathrm{number}\mathrm{of}\mathrm{plants}\\ \mathrm{in}\mathrm{each}\mathrm{scale}) \end{array}}{\begin{array}{c} \mathrm{total}\,\mathrm{number}\,\mathrm{of}\,\mathrm{plants}\times \mathrm{highest}\\ \mathrm{disease}\,\mathrm{scale} \end{array}}\times 100$$

### Assay of hydrogen peroxide generation

The H_2_O_2_ titers in all treated groups were evaluated by the method of Velikova et al. (2000), with slight modification. Initially, the H_2_O_2_ was extracted by homogenizing the dried plant cells in 0.1% trichloroacetic acid (TCA) in a ratio of 1:10 (w/v), where the clear supernatant was used as an H_2_O_2_ source. Plant leaf extract (0.5 ml) was added to 1 ml of potassium iodide (1 M), 0.5 ml of potassium phosphate buffer (10 mM, pH 7.0), and vortexed briefly. After 5 min at room temperature, the reaction absorbance was measured at 390 nm, where results were deduced using a standard curve of H_2_O_2_.

### Determination of lipid peroxidation

According to Heath and Packer (1968), lipid peroxidation was evaluated in all plant groups regarding MDA content. First, plant leaf samples were homogenized in TCA (0.1%) in a ratio of 1:5 (w/v) ratio, where the clear supernatant was used for MDA determination. Plant leaf extract (1 ml) was added to 4 ml of TCA- thiobarbituric acid solution (20% TCA supplemented with 0.5% thiobarbituric acid) and incubated for 30 min at 95°C. Subsequently, the reaction was terminated through direct cooling into ice. The developed color was measured at 600 nm. The MDA contents were deduced from the 155/mM/cm extinction coefficient.

### Determination of 2,2-diphenyl-1-picrylhydrazyl free radical scavenging activity

The free radical scavenging activity in the different treatment groups (G1–G4) was evaluated using the 2,2-Diphenyl-1-picrylhydrazyl (DPPH) method as described in Shimada et al. (1992). The scavenging rate was determined by reducing absorbance at 517 nm for 30 min in a reaction mixture that included 100 μl of plant extract (in phosphate buffer pH 7.0) and 2 ml of DPPH (0.05 M in methanol). The radical scavenging capacity results were expressed as a percentage (%) using the following equation:


(3) $$R\,a\,d\,i\,c\,a\,l\,s\,c\,a\,v\,e\,n\,g\,i\,n\,g\%=\frac{A\,0-A\,30}{A\,0}\times 100.$$

where *A*0 is the absorbance of the control and *A*30 is the absorbance of the treatment extract.

### Determination of total phenolic content

The effects of *Rhizobium* treatments upon total phenolic compounds in faba bean plants were evaluated through the Folin–Ciocalteu method according to Velioglu et al. (1998) with some modifications. First, dried plant samples (10 mg) were extracted for 24 h with 10 ml of methanol (80%) under shaking at 100 rpm. The resulting plant leaf extracts (1 mg/ml) were added (100 μl) separately to 75 μl of the Folin–Ciocalteu reagent. After 5 min, 750 μl of Na_2_CO_3_ was added and mixed. The reaction mixture was left at room temperature for 90 min, and then the UV-Vis spectrophotometer was used to measure it at 725 nm. A standard curve of gallic acid was used to figure out the TPC.

### Antioxidant enzyme assays

To evaluate the enzymes responsible for antioxidant activity in all treated groups (G1–G4), plant leave samples from each treatment group were collected, dried in liquid nitrogen, and powdered in a mortar. The enzyme contents were extracted from dried plant powder in 4 volumes of phosphate buffer (100 mM, pH 7.0), supplemented with Na-EDTA (100 mM) and polyvinylpyrrolidone (1% w/v). The clear supernatant was used as a source for enzyme activity.

### Superoxide dismutase activity

Superoxide dismutase activity was assayed in all treatment groups by the photocatalytic reduction method of nitro blue tetrazolium (NBT) chloride according to Beauchamp and Fridovich (1971) with minor modification. The reaction mixture included 100 μl of plant extract, 20 μM riboflavin, 75 μM NBT, 0.05 mM EDTA, and 13 mM methionine. The final volume was adjusted to 1.5 ml by 50 mM potassium phosphate buffer (pH 7.8). The photochemical reaction was initiated by two fluorescent lamps (15-W) for 15 min at 25°C, followed by incubation for 15 min under dark conditions. The color reduction was measured at 560 nm, where a 50% reduction in the NBT represents one unit of SOD activity.

### Catalase activity

Catalase (CAT) activity was evaluated in various treated plant groups by the H_2_O_2_ decomposition method according to Cakmak and Marschner (1992) with minor modification. Plant extracts (50 μl) were added separately to 10 mM H_2_O_2_ in phosphate buffer (25 mM, pH 7.0) to a final volume of 1 ml. The H_2_O_2_ concentration was measured at 240 nm, where the decay of 1 μmol/min of H_2_O_2_ represented one unit of CAT activity under the assay conditions.

### Ascorbate peroxidase activity

Ascorbate peroxidase activity was assayed according to the method of Nakano and Asada (1981) with slight modification as follows: 100 μl of plant enzyme extract was added to 0.25 mM ascorbic acid. The final reaction volume was adjusted to 1 ml with 25 mM phosphate buffer at pH 7.0, supplemented with 0.1 mM EDTA, and initiated by adding 10 μl of 10 mM H_2_O_2_. At 290 nm, the absorbance was measured. One unit of APX activity was equal to a 0.1 change in the reaction absorbance under the assay conditions.

### Polyphenol oxidase activity

Polyphenol oxidase activity was determined in plant groups through the quinone method described by Cho and Ahn (1999). The plant leaves (1 g) were extracted in phosphate buffer (100 mM, pH 7.0). Plant extract (500 μl) was incubated with 1 ml of quinone (50 mM in 100 mM Tris–HCl buffer pH 6.0). After setting the reaction mixture at 25°C for 10 min, the absorbance was measured at 420 nm. One unit of the enzyme activity (μM/g of fresh weight) is equivalent to increasing the absorbance by 0.001 at the assay conditions.

### Quantitative real-time PCR

The relative expression levels of four PR (*PR-1*, *PR-2*, *PR-3*, and *PR-5*) genes of faba bean were evaluated in all treatment groups through quantitative real-time PCR (qRT-PCR). Plant total RNA was extracted from leaf samples of each treatment collected at 2, 4, and 5 dpi using the RNeasy plant mini kit (QIAGEN, Germany) according to the manufacturer’s instructions and quantified by the NanoDrop UV spectrophotometer (Labtech International Ltd., Sussex, United Kingdom). The total RNA was used as a template for cDNA synthesis. As described by AbdEl-Rahim et al. (2010), DNase I-treated RNA (2 μg) was reverse transcribed to cDNA using oligo (dT) and random hexamer primers with the reverse transcriptase enzyme of Super-Script II (Invitrogen, United States). The amplified cDNA was used as a template for qRT-PCR through sets of PR primers (Table 1) with β-actin gene (housekeeping gene) to normalize the transcription levels. The qPCR reaction was conducted according to the manufacturer’s instructions of the SYBR Green PCR Master Mix (Fermentas, United States) on Rotor-Gene 6000 (QIAGEN, ABI System, United States) (Hafez et al., 2013). The relative expression level of the target gene was accurately quantified and calculated according to the 2^–ΔΔ*Ct*^ algorithm (Livak and Schmittgen, 2001). The results were expressed as relative gene expression compared to mocked-inoculated faba bean (a value of 1 is the base), where values >1 indicate gene accumulation (upregulation) and values <1 indicate the opposite (downregulation).

### Ethanol extracts preparation and high-performance liquid chromatography analysis conditions

The titer of different phenolic and flavonoid compounds in all faba bean plants was evaluated in leaf samples collected from different treatment groups. Air-dried and milled leaf samples (2 g) were extracted for 5 h at 35°C with shaking in 15 ml of ethanol (96%). Afterward, the extract was filtrated and vacuum dried, where the phenolic and flavonoid contents were analyzed through HPLC (Agilent 1260 Infinity HPLC series, equipped with a Zorbax Eclipse Plus C18 column 100 mm × 4.6 mm). The analysis was conducted using a mixture of 0.2% phosphoric acid, acetonitrile, and methanol as mobile phase, an injection sample of 20 μl, and a VWD detector at 284 nm. The standard polyphenolic compounds used were benzoic acid, caffeic acid, catechin, catechol, chlorogenic acid, cinnamic acid, ellagic acid, ferulic acid, gallic acid, kaempferol, myricetin, naringenin, *p*-coumaric acid, p-hydroxybenzoic acid, quercetin, quinol, resvertol, rosemarinic, rutin, syringic acid, and vanillic acid.

### Gas chromatography-mass spectroscopy fractionation of bacterial ethyl acetate extract

The bioactive components in the cell-free supernatant were explored and identified through gas chromatography-mass spectroscopy (GC-MS). Cell-free supernatant from a 48 h *Rhizobium* culture was mixed with ethyl acetate in a final ratio of 1:1 under shaking for 20 min. The aqueous phase was separated and dried under a vacuum. The resulting residues were analyzed through GC-MS (TRACE 1300 Series, Thermo, United States), using a mass detector in split mode with a helium gas carrier at a 1 ml/min flow rate. The injector temperature was 250°C, whereas the oven temperature was between 60 and 250°C for 20 min with a scanning time of 0.2 s and a range of 50–650 amu. Running for 53 min at 70 eV was enough to get the CFS mass spectra, which were then matched to the data already in the GC-MS library.

### Statistical analysis

All experiments were conducted in triplicates (or more), and the means (M) with standard deviations (SD) were represented as M ± SD. The significance of the represented data was analyzed through analysis of variance (ANOVA) in CoStat software using Tukey’s honest significant differences (H.S.D.) method at a probability value (*P*-Value) of ≤0.05. The significant difference between groups was shown in alphabetical order, from least important to most important (a > b > c), and the same letters showed no significant difference.

## Results

### Viral isolation and identification

**FIGURE 1:**
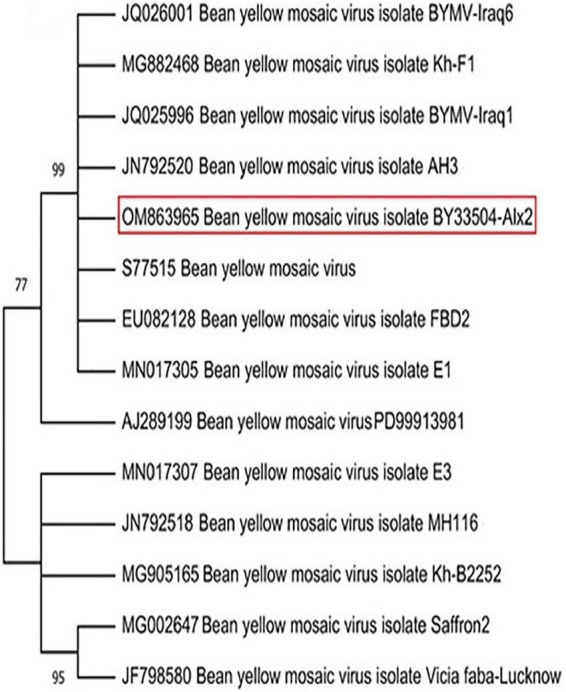
Phylogenetic tree illustrating the genetic relationship between the nucleotide sequence of coat protein gene of our *Bean yellow mosaic virus* (BYMV) isolate BY33504-Alx2 (OM863965) and other BYMV isolates obtained from GenBank. The phylogeny was created using the UPGMA statistical approach and tested using the bootstrap method with 2.000 replications.

Systemically mild to severe mosaic, green vein banding, leaf twisting, and distortion of the leaves were common symptoms in the field-collected faba bean samples. In DAS-ELISA tests, almost 91% of these samples tested positive for BYMV. A single local lesion developed on *Ch. amaranticolor* leaves at 6–7 dpi was used as a source of pure BYMV isolation. Using BYMV-CP gene primers, the RT-PCR assay gave a positive result with a 230-bp amplicon in the infected tissues. After PCR product purification and sequencing, the annotated sequence was deposited in the GenBank database under BYMV isolate BY33504-Alx2 with accession number OM863965. The NCBI-BLAST alignment and phylogenetic tree analysis showed that the BY33504-Alx2 was closely related to other BYMV isolates in GenBank, especially the Spanish isolate (Acc # JN792520), with 99% similarity (Figure 1).

### Bacterial isolation and identification

**FIGURE 2:**
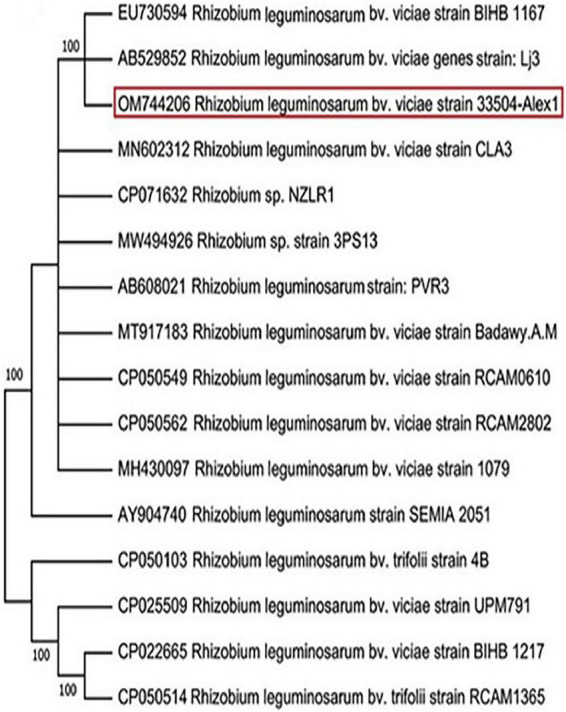
Phylogenetic tree illustrating the genetic link between 16s rRNA nucleotide sequence of our *Rhizobium leguminosarum* bv. *viciae* strain 33504-Alex1 (OM744206) and other *Rhizobium* isolates were obtained from GenBank. The phylogeny was created using the UPGMA statistical approach and tested using the bootstrap method with 2.000 replications.

Twenty-three rhizobia isolates were isolated from faba bean root nodules. All the isolates were Gram-negative, rod-shaped cells, and absorbed a small amount of Congo red, resulting in pale pink to white colonies. The rhizobia isolate exhibiting the most potent symbiotic effectiveness with faba bean plants was selected and subjected to characteristics. On the other hand, the nucleotide sequencing analysis of the amplified 16S rRNA gene revealed that the chosen bacterial isolate was identified as *R. leguminosarum* bv. *viciae*. The annotated sequence was deposited in the GenBank database under *R*. *leguminosarum* bv. *viciae* strain 33504-Alex1 with accession number OM744206. Compared to related sequences in the GenBank, the NCBI-BLAST and phylogenic tree relatedness showed that the 33504-Alex1 strain was closely related to other *R. leguminosarum* bv. *viciae* strains with 100% identity (Figure 2).

### Effect of 33504-Alex1 on faba bean growth parameters

The effects of *Rhizobium* treatment on the growth of faba bean plants were evaluated in terms of shoot and root properties. As indicated in Table 2, the plant height was remarkably reduced upon viral infection, as shown by the shoot and root length reduction in the G2 treatment compared to the control (G1), asserting the adverse impact of BYMV on faba bean growth. The soil treatment with 33504-Alex1 (G3) significantly enhanced both shoot and root length (40.31 and 25.31, respectively), representing a 1.34- and 1.65-fold increase in shoot and root length compared to the G2 treatment (30.03 and 15.31 cm, respectively). Foliar spraying of 33504-Alex1 (G4) comes next to G3 treatment in alleviating the impact of viral infection on plant growth (33.72 and 20.71 for the shoot and root length, respectively) compared to G2 treatment. The plant weight results were consistent with plant length results, where G2 treatment showed lower weights in the shoot (6.89 and 2.04 g for fresh and dry weight, respectively) and root system (5.22 and 1.27 g for fresh and dry weight, respectively) compared to the G1 group (Table 2). Compared to the G2 treatment, the G3 and G4 treatments significantly increased the fresh and dry weights of shoot and root (Table 2). On the other hand, the G4 treatment slightly boosted the growth of faba bean plants, with fresh and dry weights of shoot of 7.84 and 2.69 g and root of 6.11 and 1.62 g, respectively, though the weight gain was slightly less than the G3 treatment.

**TABLE 2:** The effect of *Rhizobium leguminosarum* bv. *viciae* strain 33504-Alex1 on faba bean growth upon infection with *Bean yellow mosaic virus* and total chlorophyll content.

Treat.	Shoot			Root			Total chlorophyll content
	Length (cm)	Fresh weight (g)	Dry weight (g)	Length (cm)	Fresh weight (g)	Dry weight (g)	
G1	35.36 ± 2.22 b	8.44 ± 1.47 b	2.89 ± 069 a	21.71 ± 1.77 b	6.91 ± 1.33 b	2.11 ± 1.02 b	39.23 ± 2.04 a
G2	30.03 ± 1.61 d	6.89 ± 1.16 d	2.04 ± 0.5 c	15.31 ± 1.38 d	5.22 ± 1.43 d	1.27 ± 1.08 d	23.95 ± 1.14 d
G3	40.31 ± 2.01 a	8.04 ± 2.49 a	3.05 ± 1.03 a	25.31 ± 2.13 a	7.67 ± 1.43 a	2.77 ± 1.25 a	35.78 ± 1.69 b
G4	33.72 ± 1.97 c	7.84 ± 1.83 c	2.69 ± 0.86 b	20.71 ± 2.21 c	6.11 ± 1.13 c	1.62 ± 1.76 c	33.68 ± 1.37 c

Each column value represents the average of five biological replicates. The mean values of each column with the same letter are not significantly different.

Furthermore, the total chlorophyll content in all treatments was analyzed directly in the fresh leaf samples (Table 2). The results indicate a significant reduction in the chlorophyll content due to viral infection as indicated in the G2 treatment (23.95 SPAD unit) with a 1.61-fold decrease (about 39%) when compared to the G1 treatment (39.23 SPAD unit). On the other hand, G3 and G4 treatments enhanced the chlorophyll content with a maximum level in the G3 group of 35.78 SPAD units, followed by the G4 group of 33.68 SPAD units (Table 2). The maximum amount of chlorophyll in the treatment groups was 35.78 SPAD units, slightly less than in the G1 treatment but 1.5 times more than in the G2 treatment.

### Effect of 33504-Alex1 on viral symptom development, disease severity, and *Bean yellow mosaic virus* inhibition index

**FIGURE 3:**
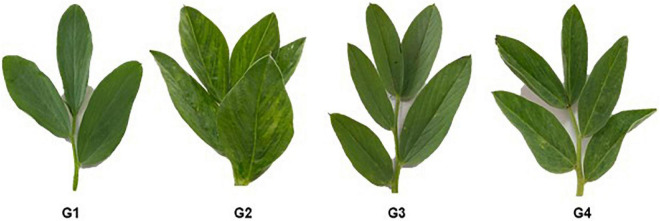
Morphological variation in faba bean leaves under *Bean yellow mosaic virus* (BYMV) challenge in different treatment groups, including G1: control plants, G2: BYMV-infected plants, G3: plants of soil treated with a *Rhizobium* isolate 4 days before BYMV inoculation, G4: plants treated by foliar spraying of culture filtrate, 24 h before inoculation with BYMV.

Under greenhouse conditions, the G2 treatment developed mosaic signs at 10 dpi. At 12 dpi, severe mosaic and green vein banding are clearly visible (Figure 3). Interestingly, the application of 33504-Alex1 in either soil (G3 treatment) or foliar application (G4 treatment) was associated with a delayed symptom appearance of approximately 3 days with mild symptoms at 15 dpi, compared to G2 treatment (Figure 3). No symptoms were observed during the G1 treatment. The G2 treatment reported 100% of disease incidence with a DS of 91.34% (Table 3). However, G3 treatment plants significantly reduced disease incidence and severity to 40% (6 infected plants/15 plants) and 15.46%, respectively. Moreover, G4 treatment exhibited 53.33% (8 infected plants/15 plant) disease incidence and 21.38% DS (Table 3). Furthermore, treatment with 33504-Alex1 in soil (G3) or foliar (G4) considerably increases the inhibition index of BYMV in the treatment of faba bean plants. As presented in Table 3, the highest ELISA value (1.85) was recorded in the G2 treatment, followed by the G4 and G3 treatments, with ELISA values of 0.35 and 0.49, respectively.

**TABLE 3:** Effect of *Rhizobium* treatments and *Bean yellow mosaic virus* (BYMV) on faba bean disease incidence, disease severity, and virus accumulation level at 21 dpi.

Treatment	Disease incidence (%)	Disease severity (%)	ELISA values*
G1	00 ± 0.00 d	00.00 ± 0.00 d	0.10 ± 0.05 d
G2	100 ± 0.00 a	91.34 ± 2.68 a	1.85 ± 0.07 a
G3	40.00 ± 0.13 c	15.46 ± 1.31 c	0.35 ± 0.02 c
G4	53.33 ± 0.16 b	21.38 ± 1.53 b	0.49 ± 0.03 b

*ELISA absorbance at 405 nm, positive result = double the healthy absorbance. Each column value represents the average of five biological replicates. The mean values of each column with the same letter are not significantly different.

### Oxidative stress markers, free radical quenching activity, and total phenolic compounds estimation

**FIGURE 4:**
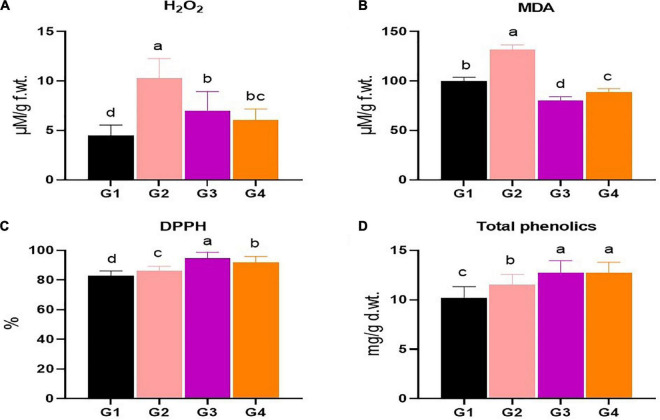
The histogram shows the evaluation of phenolic compounds and various oxidative stress markers in faba bean plants under the *Bean yellow mosaic virus* (BYMV) challenge, including **(A)** total phenolic compounds, **(B)** free radical quenching activity (DPPH), **(C)** hydrogen peroxides (H_2_O_2_), and **(D)** malondialdehyde (MDA). All compounds were evaluated in the treatment groups: G1: control plants, G2: BYMV-infected plants, G3: plants of soil treated with a *Rhizobium* isolate 4 days before BYMV inoculation, G4: plants treated by foliar spraying of culture filtrate, 24 h before inoculation with BYMV. The columns reflect the mean of five biological replicates, while the bars represent the standard deviation (±SD). Columns with the same letter meaning do not differ significantly.

The two oxidative stress markers, H_2_O_2_ and MDA, were evaluated in the four faba bean treatment groups in terms of content (Figure 4). The H_2_O_2_ level was considerably elevated in the G2 treatment plants, up to 10.29 μM/g f.wt., representing about a 2.3-fold increase compared to the G1 treatment (4.48 μM/g f.wt.), as indicated in Figure 4A. Soil application of 33504-Alex1 significantly diminished the H_2_O_2_ in the G3 group (6.99 μM/g f.wt.). The foliar application of 33504-Alex1 showed the maximum H_2_O_2_ reduction with about a 59% reduction from the H_2_O_2_ level reported due to viral infection in the G2 group (Figure 4A). Similarly, the results of MDA indicate upregulated high lipid peroxidation in the G2 group (132.14 μM/g f.wt.) compared to the G1 treatment (99.98 μM/g f.wt.), which is about 32% higher (Figure 4B). On the other hand, the G3 and G4 treatments showed significant reductions in MDA levels, indicating lower lipid peroxidation (80.59 and 89.13 μM/g f.wt., respectively) compared to the G2 group. Figure 4B shows that the MDA level was lower in G3 and G4 than in G1. For G3, the reduction was 20%, and for G4, it was 11%.

The free radical quenching activity in the different treatment groups (G1–G4) was evaluated based on the DPPH approach. As shown in Figure 4C, the results showed that the free radical scavenging was slightly more active in the G2 group (86.28%) than in the G1 group (83.05%). The G3 and G4 treatments significantly increased free radical scavenging activity, with the G3 treatment reporting the highest level at about 94.67%, representing a 10 and 14% increase over the G2 and G1 treatment groups, respectively (Figure 4C). Regarding the phenolic compounds, the results revealed a slight increase in the total phenolic content in the G2 treatment, which could be attributed to the systemic defense of faba bean plants against viral infection (Figure 4D). Figure 4D shows that treatment with 33504-Alex1 significantly increased the total phenolic content in the G3 (12.76 mg/g d.wt.) and G4 (12.71 mg/g d.wt.) groups by about 25% compared to the G1 (10.17 mg/g d.wt.) group.

### Antioxidant enzyme assays

**FIGURE 5:**
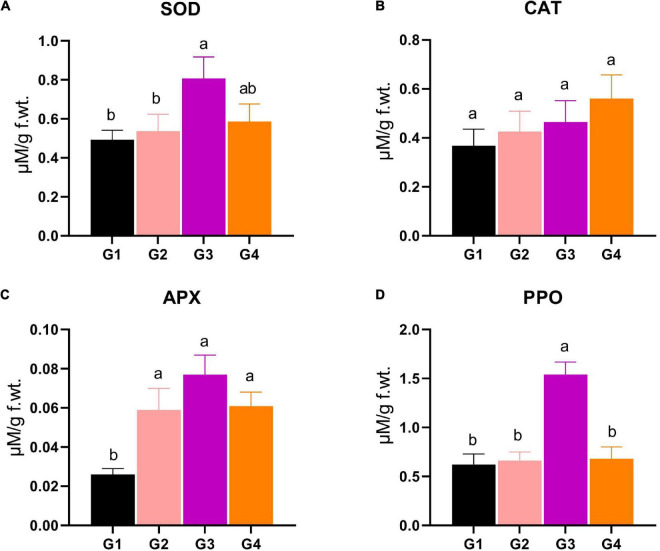
Evaluation of various antioxidant enzymes production, including **(A)** superoxide dismutase (SOD), **(B)** catalase (CAT), **(C)** ascorbate peroxidase (APX), and **(D)** polyphenol oxidase (PPO) in *Bean yellow mosaic virus* (BYMV)-infected fab bean plants: G1: control plants, G2: BYMV-infected plants, G3: plants of soil treated with a *Rhizobium* isolate 4 days before BYMV inoculation, G4: plants treated by foliar spraying of culture filtrate, 24 h before inoculation with BYMV. The columns reflect the mean of five biological replicates, while the bars represent the standard deviation (±SD). Columns with the same letter meaning do not differ significantly.

Four important antioxidant enzymes, such as SOD, CAT, APX, and PPO, were measured to find out how much antioxidant activity each treatment group had. For SOD, the G3 and G4 treatments revealed significantly superior SOD production (0.807 and 0.586 μM/g f.wt., respectively) compared to the SOD level in the G1 and G2 treatments (0.493 and 0.537 μM/g f.wt.) (Figure 5A). The SOD level of the G3 treatment represents about a 1.5-fold increase compared to the control (Figure 5A). Regarding CAT, the statistical analysis revealed that no significant changes were reported between different treatments (Figure 5B). Concerning the APX activity, the data represented a significant increase in the enzyme level in the G2, G3, and G4 treatments (0.059, 0.077, 0.061 μM/g f.wt., respectively), compared to G1 (0.026 μM/g f.wt.), asserting the vital role of APX in the protection against BYMV infection (Figure 5C). The PPO activity showed that the G3 treatment exhibited maximum activity (1.54 μM/g f.wt.), representing 2.5- and 2.3-fold significant increases compared to G1 and G2, respectively (Figure 5D).

### Pathogenesis-related genes expression levels

**FIGURE 6:**
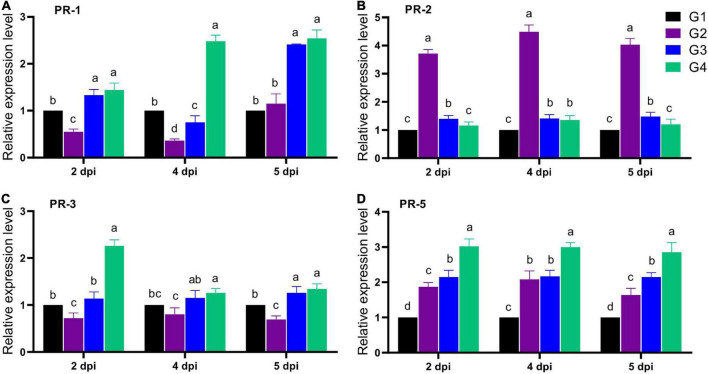
The effect of *Rhizobium* cells and supernatant treatment upon the relative expression levels of four pathogen-related genes after 2, 4, and 5 dpi of *Bean yellow mosaic virus* (BYMV) infection for faba bean plants, including **(A)** PR-I, **(B)** *PR-2*, **(C)** *PR-2*, and **(D)** *PR-5* genes. G1: control plants, G2: BYMV-infected plants, G3: plants of soil treated with a *Rhizobium* isolate 4 days before BYMV inoculation, G4: plants treated by foliar spraying of culture filtrate, 24 h before inoculation with BYMV. The columns reflect the mean of five biological replicates, while the bars represent the standard deviation (±SD). Columns with the same letter meaning do not differ significantly.

The expression of four faba bean PRs (*PR-1*, *PR-2*, *PR-3*, and *PR-5*) was evaluated in all treatment groups after 2, 4, and 5 dpi (Figure 6). For *PR-1* expression level, the G2 treatment significantly reduced its transcriptional level at 2 and 4 dpi, with a maximum reduction level at 4 dpi, reporting a relative transcriptional level of 0.36-fold change lower than the G1 treatment. Interestingly, soil treatment with 33504-Alex1 (G3) significantly enhanced the *PR-1* overexpression from 2 dpi, with a maximum level detected at 5 dpi, representing a 2.41-fold increase compared to the G1 treatment (Figure 6A). On the other hand, the foliar spraying of 33504-Alex1-CF was the most potent upregulator for the *PR-1* gene, with relative expression levels of 1.44, 2.48, and 2.54-fold change higher than control at 2, 4, and 5 dpi, respectively (Figure 6A). The results indicated nearly equal *PR-1* induction potential for G3 and G4 at 2 and 5 dpi. However, the 4 dpi results showed that foliar application of 33504-Alex1-CF increased the *PR-1* expression level. This means that spraying 33504-Alex1-CF has a faster effect on increasing plant resistance by over-activating the *PR-1* gene.

The second evaluated gene was the *PR-2* gene. The G2 treatment showed significant up-regulation in the *PR-2* expression level with a 3.72- and 4.03-fold increase in the gene expression at 2 and 5 dpi. The maximum level was at 4 dpi, with a relative expression level of 4.49-fold higher than the control (Figure 6B). Compared to the G2 treatment, the G3 and G4 treatment groups showed a significant reduction in PR-2 expression levels with values nearly the control (Figure 6B). Treatment with 33504-Alex1 CF (G4) was slightly better at reducing *PR-2* than the G3 treatment. The G3 treatment showed a slight increase in PR-2 expression levels, about 1.40-fold higher than the control at all dpi (Figure 6B). Regarding *PR-3*, the qPCR results indicated a significant reduction in *PR-3* gene expression in the G2 treatment by about 26% at all dpi compared to the G1 treatment (Figure 6C). At 2 dpi, the foliar application of 33504-Alex1-CF significantly increased the *PR-3* expression level by 2.26-fold compared to the G1 treatment (Figure 6C). At 5 days post-infection (dpi), the PR-3 transcript levels were about 1.3 times higher in the G3 and G4 groups than in the control group (Figure 6C). Like the *PR-3* transcript profile, the G4 treatment exhibited the highest transcriptional levels of *PR-5* with relative expression levels of 3. 02-, 3. 01-, and 2.85-fold change higher than control at 2, 4, and 5 dpi, respectively (Figure 6D). Also, the G2 (1. 87-, 2. 08-, and 1.64-fold) and G3 (2. 15-, 2. 17-, and 2.15-fold) treatments significantly increased PR-5 transcriptional levels at 2, 4, and 5 dpi, respectively (Figure 6D).

### Evaluation of phytochemical constituents of the different treatment groups

**FIGURE 7:**
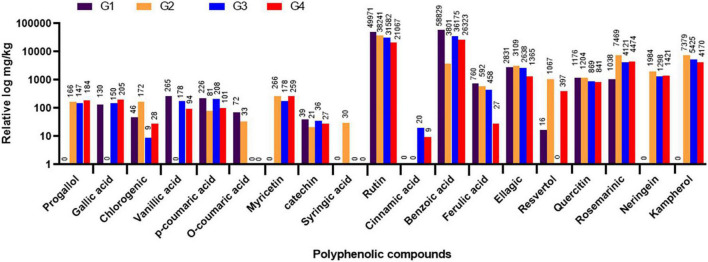
The high-performance liquid chromatography (HPLC) analysis of different polyphenolic and flavonoids in leaf samples of faba bean as detected at 20 dpi. G1: control plants, G2: *Bean yellow mosaic virus* (BYMV)-infected plants, G3: plants of soil treated with a *Rhizobium* isolate 4 days before BYMV inoculation, G4: plants treated by foliar spraying of culture filtrate, 24 h before inoculation with BYMV. The results were represented with the relative log, where compound values were represented above each column.

The variations in the phytochemical constituents within the different faba bean treatment groups (G1-G4) were evaluated in the leaf samples through HPLC after 20 dpi. The results showed that the other treatment groups had different amounts of polyphenolic compounds. The total amount of the 19 polyphenolic compounds found was 115,405 mg/kg for G1, 65,621.8 mg/kg for G2, 83,499.3 mg/kg for G3, and 60,998.9 mg/kg for G4. The major detected compounds were benzoic acid, rutin, ellagic, rosemarinic, and quercetin, with more than 1,000 mg/kg (Figure 7). The HPLC analysis revealed that the four flavonoid compounds, pyrogallol, neringein, myricetin, and kaempferol, were induced after BYMV inoculation (Figure 7). The fact that this compound was found in all virus-infected groups (G2, G3, and G4) but not in the control group showed that it was part of a plant’s defense against virus infection. On the other hand, the suppressor activity of BYMV inhibited the accumulation of gallic and vanillic acids in the G2 treatment. At the same time, they were detected in the G1 control group (130.42 and 265.67 mg/kg, respectively) and the treatment groups G3 and G4 (150.49 and 205.17 for gallic acid and 178.68 and 94.73 for vanillic acid in both groups, respectively). Interestingly, the application of 33504-Alex1 led to the production of cinnamic acid, which was different from the G1 and G2 treatments (Figure 7). Moreover, syringic acid was exclusively detected in the G2 treatment.

### Evaluation of the bioactive components in the 33504-Alex1-CF through gas chromatography-mass spectroscopy analysis

**FIGURE 8:**
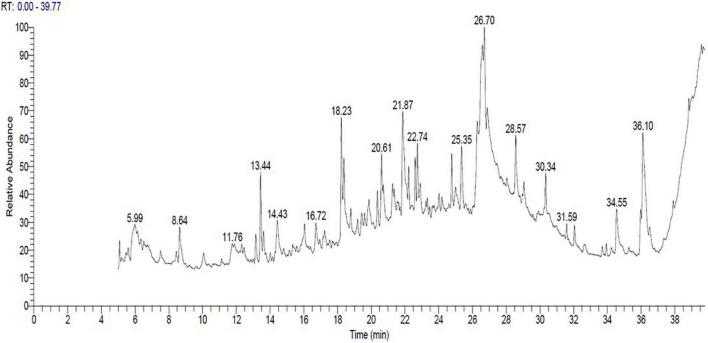
The cell-free supernatant chromatogram for *Rhizobium leguminosarum* bv. *viciae* strain 33504-Alex1 ethyl acetate extract constituents as elucidated with gas chromatography-mass spectrometry (GC-MS).

The bioactive components of 33504-Alex1-CF were elucidated in the ethyl acetate extracted supernatant through GC-MS analysis. Figure 8 shows that the result showed that there were several compounds because there were many peaks at different retention times (RT). In Table 4, 17 detected components are listed. These include 3,6-octadecadiynoic acid, methyl ester; octanoic acid, ethyl ester; 4-trifluoroacetoxytetradecane; 5-heptenoic acid, 6-methyl-4-[(4-ethylphenyl) sulfonyl]; methyltricyclo[6.5.2(13,14)0.0(7,15)]pentadeca-1,3,5,7,9,11,13-heptene; 1-hexadecanol, 2-methyl-; 1,3,5-triazine-2,4-diamine, 6-chloro-N-ethyl-; (3-methyl-1,4-diphenylbicyclo[2.2.0]hex-2- yl)methanol; isochiapin B; tetraneurin-A-Diol; hexadecanoic acid 2,3-dihydroxypropyl ester; oleic acid; dotriacontane; 2,2,3,3,4,4 hexadeutero octadecanal; 9,12,15-octadecatrienoic acid; 4,7-octadecadiynoic acid, methyl ester; diisooctyl phthalate were detected at different RT. The GC-MS analysis also revealed the presence of retinal (aldehyde form of vitamin A) at RT of 21.26 min and several bioactive fatty acids, including oleic acid at RT of 26.95, *cis*-11-eicosenoic acid at RT of 26.28 min, and hexadecanoic acid 2,3-dihydroxypropyl ester at RT of 26.71 min.

**TABLE 4:** The constituents of *Rhizobium leguminosarum* cell-free supernatant as elucidated through gas chromatography-mass spectrometry (GC-MS) analysis.

RT	Compound name	Area %	Molecular formula	Molecular weight
5.99	3,6-Octadecadiynoic acid, methyl ester	4.56	C_19_H30O_2_	290
8.64	Octanoic acid, ethyl ester	2.42	C_10_H_20_O_2_	172
13.44	4-Trifluoroacetoxytetradecane	3.75	4C_16_H_29_F_3_O_2_	310
14.43	5-Heptenoic acid, 6-methyl-4-[(4-ethylphenyl) sulfonyl]	2.06	C_15_H_20_O_4_S	296
16.72	Methyltricyclo[6.5.2(13,14)0.0(7,15)]pentadeca-1,3,5,7,9,11,13-heptene	1.27	C_16_H_14_	206
18.23	1-Hexadecanol, 2-methyl-	5.83	C_17_H_36_O	256
20.61	1,3,5-Triazine-2,4-diamine,6-chloro-*N*-ethyl-	3.47	C_5_H_8_ClN_5_	173
21.87	(3-Methyl-1,4-diphenylbicyclo[2.2.0]hex-2-yl)methanol	5.78	C_20_H_22_O	278
22.74	Isochiapin B	3.05	C_19_H_22_O_6_	346
25.35	Tetraneurin-A-diol	3.52	C_15_H_20_O_5_	280
26.70	Hexadecanoic acid 2,3-dihydroxypropyl ester	7.97	C_19_H_38_O_4_	330
26.70	Oleic acid	7.35	C_18_H_34_O_2_	282
28.57	Dotriacontane	2.80	C_32_H_66_	450
30.34	2,2,3,3,4,4 Hexadeutero octadecanal	1.76	C_18_H_30_D_6_O	274
31.59	9,12,15-Octadecatrienoic acid	0.67	C_27_H_52_O_4_Si_2_	496
34.55	4,7-Octadecadiynoic acid, methyl ester	2.13	C_19_H_30_O_2_	290
36.10	Diisooctyl phthalate	7.81	C_24_H_38_O_4_	390

## Discussion

Legume crops are an essential source of food, nutrition, and industrial materials for humans, animals, and the environment (Moawad et al., 2004). Many phytohormones, such as brassinosteroids, cytokinins, enzyme 1-aminocyclopropane-1-carboxylate (ACC) deaminase, ethylene, gibberellins, IAA, jasmonates, lipo-chito-oligossacharide Nod factors, lumichrome, rhizobitoxine, and riboflavin, were synthesized and released during the symbiotic interaction process between rhizobia and legumes (Jaiswal et al., 2021). Such compounds can directly or indirectly stimulate plant growth and may promote plant adaptation to various stresses (Purwaningsih et al., 2021). *Rhizobium* is a natural PGPR in the rhizosphere used in farming to enhance legume crops’ growth through mutually beneficial relationships (Allito et al., 2021; Boivin et al., 2021). This way, finding new biocontrol agents that are safe for the environment, control plant viruses, and fix nitrogen is a top priority for food security.

Under greenhouse conditions, *R. leguminosarum* bv. *viciae* strain 33504-Alex1 was applied to alleviate the BYMV infection in faba beans. The results showed that the growth parameters of faba beans significantly improved when 33504-Alex1 was applied to the soil (G3 treatment) or the leaves (G4 treatment). The soil treatment had a more considerable effect than the foliar treatment. The importance of 33504-Alex1 in plant growth enhancement can be attributed to PGPR’s direct effect on increasing nutrient availability, production of plant growth-promoting phytohormones, and water uptake (Elhelaly, 2022; Rizvi et al., 2022). The total chlorophyll content was reduced in the BYMV-challenged non-treated plants (G2 treatment), which is in accordance with Sofy et al. (2020), who reported a decrease in the chlorophyll by about 43% in faba bean plants under the BYMV challenge compared to control. The chlorophyll reduction could be attributed to the direct adverse effect of BYMV on chloroplast functions (Radwan et al., 2008). Interestingly, the application of 33504-Alex1 significantly increased chlorophyll content by 49.39 and 40.62% in G3 and G4, respectively, compared to the G2 treatment. On the other hand, the results showed that 33504-Alex1 could protect faba bean plants from BYMV by lowering disease incidence (up to 60%), decreasing its severity (up to 83%), and increasing inhibition index (up to 81%) in treated plant tissues. These results were in harmony with those obtained by Elhelaly (2022), who showed that the treatment of faba bean seeds with *R. leguminosarum* significantly decreased the disease incidence of BYMV infection compared to untreated seeds. The significant increase in the inhibition index of BYMV confirmed the protective efficacy of 33504-Alex1 against BYMV infestation. Thus, using 33504-Alex1 could boost the host’s innate immune system or cause SAR, preventing BYMV from spreading or inhibiting its replication.

The elevation of the two oxidative stress markers, H_2_O_2_ and MDA, was consistent with the common BYMV-plant infection feature combined with a high titer of reactive oxygen species (Radwan et al., 2010; Sofy et al., 2020). Lipid peroxidation (MDA) directly indicates membrane disruption and deterioration of the plant cells due to intolerable oxidative stress (Loreto and Velikova, 2001; Alché, 2019). Both markers have increased in the G2 group, whereas they have been significantly reduced in the G3 and G4 groups. The results were similar to Hamzah et al. (2021), who reported the induction of MDA in faba bean plants upon inoculation with BYMV. The positive effects of rhizobia are mediated by creating various compounds and enzymes directly or indirectly induced by rhizobia and plants during nodule formation (Jaiswal et al., 2021). As a result, the 33504-Alex1 application may increase phenolic compounds and free radicals while activating the non-enzymatic pathway to reduce the risk of oxidative stress. The results were similar to those reported by Hamzah et al. (2021), who reported that the inoculation of faba bean plants with rhizobia was associated with an increase in total phenolic content compared to non-treated plants. In the same regard, the antioxidant enzyme results revealed a slight increase in all enzymes under the current study in plants under BYMV challenge (G2), which could be related to the activation of the faba bean defense system as an initial response to BYMV infection. However, the antioxidant enzyme levels in G2 were insufficient to alleviate the adverse effects of the reactive oxygen species on the plant, as indicated by the lower growth parameters and higher oxidative stress markers in the G2 treatment results. The generation of reactive oxygen species is well documented under several viral infections due to the impairment of the electron transport chain (Chandra et al., 2015; Sofy et al., 2020); hence, the overproduction of the antioxidant enzyme is crucial for mitigating oxidative stress. When 33504-Alex1 was used, the antioxidant enzymes’ production increased drastically, and soil treatment was much better than foliar treatment in all enzyme assays.

Pathogenesis-related genes encode essential plant defense proteins in response to several biotic and abiotic stressors. The expression levels of four PR genes were analyzed to investigate the molecular processes underlying faba bean resistance to BYMV infection following treatment with 33504-Alex1. The *PR-1* expression level was significantly reduced due to viral infection, with up to a 64% decrease at 4 dpi compared to the G1 treatment. Notably, the G3 or G4 treatments significantly increased *PR-1* overexpression, with a maximum level detected at 5 dpi. The results are consistent with the reported role of the *PR-1* gene in mitigating BYMV and other mosaic viruses’ infection (Sofy et al., 2020; Abdelkhalek et al., 2021). On the contrary, the *PR-2* gene was significantly elevated in the G2 treatment, with a maximum relative expression level of 4.49-fold increase at 4 dpi compared to the G1 treatment. The *PR-2* gene encodes a β-1,3-glucanase protein, an essential enzyme in cell-to-cell translocation and signaling. This enzyme hydrolyzes the callose deposits between plant cells, so its overexpression is necessary for most viruses to facilitate cell-cell viral translocation (Kawakami et al., 2004; Oide et al., 2013; Otulak-Kozieł et al., 2018). Interestingly, G3 and G4 treatments revealed a significant reduction in *PR-2* expression levels, nearly to the G1 level. The foliar application of 33504-Alex1-CF on the leaves was the most effective way to reduce *PR-2* expression, with a 3.2-fold drop compared to the G2 treatment.

Furthermore, the *PR-5* (Thaumatin-like proteins) was upregulated upon treatment with 33504-Alex1. The results are consistent with the reported role of *PR-5* in improving the plant’s resistance to numerous infections (Zhang et al., 2017). In the same line with *PR-1* and *PR-3* gene results, the 33504-Alex1-CF foliar spraying was most effective in inducing *PR-5* accumulation, which is composed of the accumulation of PR proteins in the infected and surrounding plant tissue. Hence, foliar treatment is more logical for infected plants (Chandrashekar et al., 2018). The *PR-1* and *PR-5* gene expression are regulated by salicylic acid (SA), which is the primary mediator of the systemic acquired resistance (SAR) mechanism (Breen et al., 2017; Wang et al., 2021). Thus, the results showed that 33504-Alex1 could boost SA-mediated SAR in faba bean when infected with BYMV. Ali et al. (2018) found that jasmonic acid (JA) and ethylene are the main mediators of induced systemic resistance. JA and ethylene control the PR-3 gene that codes for chitinase activity. In the current study, the *PR-3* was significantly activated in G4 treatment at 2 dpi. According to the TMV infection study (Zhu et al., 2014), the slight induction of JA at the beginning is important for the later buildup of SA. Despite its role in SA accumulation, evidence for JA’s direct participation in plant resistance to viral challenges is inconclusive (Oka et al., 2013; Zhao and Li, 2021). The *PR-3* gene upregulation is widely reported as a part of plant resistance under fungal infection (Eslahi et al., 2021; Heflish et al., 2021), with a limited role in plant viral infection (Abo-Zaid et al., 2020). This could explain why its level dropped in the BYMV-challenged plants at 4 and 5 dpi.

The HPLC analysis demonstrated that the four flavonoid compounds, namely, pyrogallol, neringein, myricetin, and kaempferol, were induced following BYMV inoculation. Kaempferol, neringein, and myricetin are powerful antioxidants, so they could be made more during viral infection to help with the higher oxidative stress (Marín et al., 2018). It was reported that myricetin could directly interact with viral glycoprotein D to prevent its adsorption and membrane fusion to host cells, thereby inhibiting viral infection and replication (Li et al., 2020). Myricetin reportedly inhibited Zika virus replication and African swine fever virus protease (Jo et al., 2020; Zou et al., 2020). Kaempferol is one of the important members of the flavonol subgroup of flavonoids. It is a critical component of the auxin-dependent defense response that prevents plant viruses from spreading systemically. This defense response is engaged before the well-known SA-dependent defense response (Likic̀ et al., 2014). These compounds were detected only in BYMV-inoculated plants, not control plants. This suggests that they play a big role in plant defense against viruses. Interestingly, gallic acid and vanillic acid were suppressed in the G2 treatment, which could be attributed to their role as natural polyphenolics with reported antioxidant, antiviral, and antibacterial activities (Sethupathy et al., 2017; Bai et al., 2021). Gallic acid revealed excellent antibacterial and antiviral activity against *Salmonella* and herpes viruses (Shahidi and Yeo, 2018). Based on the findings, we hypothesized that these two compounds, gallic and vanillic acids, may have antiviral activity against BYMV infection. However, more research is needed to confirm this hypothesis. Benzoic acid is an important building block for plants’ phytohormones, enzymes, and cofactors (Widhalm and Dudareva, 2015). Benzoic acid and rutin were the most detected polyphenolic compounds in all treatments. Interestingly, the application of 33504-Alex1 resulted in the synthesis of cinnamic acid, which was distinct from the G1 and G2 treatments. Moreover, syringic acid was exclusively found in the G2 treatment.

The GC-MS analysis indicated several bioactive molecules, including 17 compounds of polyphenolic, flavonoids, and fatty acids. Isochiapin-B was among the most detected compounds in the 33504-Alex1-CF and is a volatile terpenoid compound with several biological activities. Isochiapin B was detected in the *Achillea fragmmentissma* and *Citrus aurantium* plant extracts with potent anti-inflammatory, antimicrobial, and antioxidant activities (Elsharkawy, 2016; Değirmenci and Erkurt, 2020). Hexadecanoic acid 2,3-dihydroxypropyl ester (known as palmitate) is a biologically active fatty acid with reported antitumor activity against human leukemia cells, with no side effects or cytotoxicity to normal human dermal fibroblast (Al-Wahaibi et al., 2020). It was reported that the broad-spectrum antimicrobial activity of *Conocarpus lancifolius* leaf extract and its antitumor activity against breast cancer might be attributed to the presence of hexadecanoic acid, 2,3-dihydroxypropyl ester (Moni et al., 2021). Furthermore, oleic acids are biologically active monounsaturated fatty acids with reported antibacterial, antioxidant, and antiviral activities (Dilika et al., 2000; Wei et al., 2016; Zhao et al., 2017). Dotriacontane and tetraneurin-A-diol were also found in the GC-MS profile. Dotriacontane has high antimicrobial and antioxidant activities (Asong et al., 2019), while tetraneurin-A-diol has pesticide activity and has been found in many plants and microbial extracts (Abdelhamid et al., 2015). As a result, the GC–MS analysis indicated the existence of many active compounds with different biological activities in the 33504-Alex1-CF. The current study indicated that 33504-Alex1-CF has considerable antiviral activity against BYMV and growth-stimulating effects on faba bean plants. Consequently, 33504-Alex1-CF may be effective as a preventative biocontrol agent against plant viral infection. However, additional research is required to confirm potential field applications.

## Conclusion

Under experimental greenhouse conditions, the *R. leguminosarum* bv. *viciae* strain 33504-Alex1 isolated from faba bean root nodules seems to be a promising inducer for systemic resistance in faba bean against challenge BYMV infection. Compared to non-treated faba bean plants, the application of 33504-Alex1 in either soil or foliar application was associated with lowering disease incidence (up to 60%), decreasing DS (up to 83%), and increasing inhibition index (up to 81%), as well as promoting growth and increasing the total chlorophyll content. It also reduced H_2_O_2_ and MDA, boosted DPPH and total phenolic content, and antioxidant enzymes and PR transcription levels. Moreover, the most common phenolic compounds were found to be activated and build up, except for gallic and vanillic acids, which were completely turned off in non-treated plants. These results could be very important since they may offer a simple, environmentally safe, and economically accepted means to protect faba bean plants from BYMV infection. However, additional studies are needed to confirm these results under field conditions.

## Data availability statement

The datasets presented in this study can be found in online repositories. The names of the repository/repositories and accession number(s) can be found in the article.

## Author contributions

AA, HE-G, and SB designed the research, wrote the manuscript, performed the experiments, and analyzed the data. AA-A, VM, HM, ME, and HY helped with editing and provided suggestions for the experiments. All authors contributed to the article and approved the submitted version.

## Footnotes

1
http://blast.ncbi.nlm.nih.gov/Blast.cgi

2
http://www.ncbi.nlm.nih.gov
